# Patterns of cancer in Wolaita Sodo University Hospital: South Ethiopia

**DOI:** 10.1371/journal.pone.0274792

**Published:** 2022-10-06

**Authors:** Yitbarek M. Kibret, Yohannes A. Leka, Natnael F. Tekle, Wondemagegnehu Tigeneh

**Affiliations:** 1 Oncology Department, Yekatit 12 Hospital Medical College, Addis Ababa, Ethiopia; 2 Pathology Department, Wolaita Sodo University Teaching Referral Hospital, Wolaita Sodo, Ethiopia; 3 Internal Medicine Department, Medical Faculty, Addis Ababa University, Addis Ababa, Ethiopia; 4 Oncology Department, Medical Faculty, Addis Ababa University, Addis Ababa, Ethiopia; University of California Irvine, UNITED STATES

## Abstract

**Background:**

Variations in cancer occurrence between populations in different places are expected because of many factors. In Ethiopia there is no national cancer registry and here we are reporting the pattern of cancer in Wolaita Sodo University hospital located in Southern region of Ethiopia with catchment area of over ten million peoples.

**Methodology:**

A retrospective record analysis of all pathologically confirmed malignancies from January 2021 up to June 2021. Data was filtered and descriptive analysis was done using IBM SPSS version 22 (Chicago IL USA).

**Result:**

In the Wolaita Sodo University Teaching Referral Hospital during the first six months of 2021, out of 1,810 histopathologically tested samples 19.5% (354) were confirmed malignant cases. Among 354 patient samples, most of them (62.4%) were in females and the rest (37%) found to be in males. The age pattern shows occurrence of 336 (95%) cases in adults and 18 (5%) cases in children. Breast cancer, soft tissue sarcomas, cancer of uteri cervix, non melanomatous skin cancer, and non hodgkin lymphomas were the five top common cancers of all age groups. In adult population, breast cancer, soft tissue sarcomas, and cancer of uteri cervix are the most common. In children of age less than 14 years non hodgkin lymphomas, soft tissue sarcomas and bone sarcomas were the three top cancers. Breast cancer, cancer of uteri cervix and soft tissue sarcomas are found to be the commonest cancers in females. On the other hand, soft tissue sarcomas, non melanomatous skin cancers and Non Hodgkin lymphomas, are the three top commonest cancers in males.

**Conclusion:**

Based on our current study cancer is one of the common finding from histopathology samples analyzed at the hospital and the pattern of cancer was similar to those reported in other regions of the country as well as neighboring countries. However, Comprehensive demographic and clinical data using population or facility-based cancer registry is required to get better information. Additionally, our finding of higher proportion of soft tissue sarcomas both in males and females of all age groups in this region is disparate and requires further investigation.

## Introduction

The burden of cancer incidence and mortality is rapidly growing worldwide and has became an important barrier to increasing life expectancy [1, 2]. GLOBOCAN 2020 has predicted the global cancer burden to be 28.4 million cases in 2040, which is a 47% rise from 2020 [1]. However, the larger increase is expected in transitioning (64% to 95%) versus transitioned (32% to 56%) countries, thus making cancer an increasing problem for low- and middle-income countries in the sub-Saharan Africa region like Ethiopia [1]. Worldwide, an estimated 19.3 million new cancer cases (18.1 million excluding nonmelanoma skin cancer) occurred in 2020. Overall, Female breast cancer is the most commonly diagnosed cancer, with an estimated 2.3 million new cases (11.7%), followed by lung (11.4%) and colorectal cancer (10.0%) [3]. In males, Lung cancer (14.3%), prostate cancer (14.1%) and colorectal cancer (10.6%) are top three while in females, breast cancer (24.5%), colorectal cancer (9.4%) and lung cancer (8.4%) are the commonest [3].

Since cancer is a complex and diverse disease, its patterns of incidence vary based on differences in underlying cancer risk factors such as environmental and lifestyle factors [4]. Studies suggest cancer in economically transitioning countries is on the rise caused by a rapid population growth, higher life expectancy and adoption of unhealthy lifestyles as well as changes in reproductive patterns [4, 5]. In sub saharan Africa a total number of 801,392 cases are estimated to occur in 2020, and breast cancer (16.8%), cancer of cervix uteri (10.6%) and prostate cancer (8.4%) were the three commonest cancers overall [1]. In females, breast cancer (27.3%), cancer of cancer of cervix uteri (23.3%) and colorectal cancer (4.6%) were the commonest; While in males, prostate cancer (23.6%), cancer of the liver (7.5%) and colorectal cancer (7.1%) were the leading types [1]. Although the incidence of caner is rapidly rising in this part of the world much attention has been on infectious disease control. Unfortunately, the infectious diseases coexist with the emergence of the non-communicable diseases such as cancer and the mortality due to cancer is much higher than the commonest existing infectious diseases on the region [4]. Thus, cancer is huge challenge for the health care systems of this region of Africa and a severe force to be dealt with.

Similar to other sub-Saharan countries, the incidence of cancer is rising in Ethiopia too, with annual estimation of about 77,352 new cancer cases in 2020 alone [1]. However, since there is no national caner registry such estimates of cancer burden in the country are made on basis of extrapolations of data from the single Addis Ababa population based cancer registry. The first data from Addis Ababa population based cancer registry shows Cancers of the breast (31.5%) and cervix (14.1%) are the two most common cancers among females, while colorectal cancers (10.6%) and non-Hodgkin lymphomas (10.2%) are the most common cancers among males [6]. On the other hand a study on patterns of cancer at University of Gondor hospital in Northern Ethiopia reported Cervical cancer, breast cancer and lymphomas to be the commonest cancers in females whereas, lymphomas, head and neck squamous cell carcinomas (HNSCC), and colorectal cancer to be the three top commonest cancers in males [7].

Except in the past three years, cancer treatment in Ethiopia was confined at Tikur Anbassa hospital which is located in the capital city Addis Ababa. As part of cancer treatment service expansion into regional hospitals, the Wolaita Sodo University hospital located in southern part of Ethiopia commenced cancer treatment service on Jan, 2021. The hospital service for cancer treatment was limited to surgery as well as endocrine therapy for breast cancer before establishment of the oncology department on January 2021. The hospital serves as a referral center for four district hospitals and over six million people. There is no published data on pattern of cancer in this part of the country and incidence reports extracted from other cancer treatment institution may not truly show the actual pattern in the population of this part of the country with a different cultural and ethnic make-up. Taking this knowledge gap into consideration, we assessed the pattern of cancer seen during the first six months of initiation of cancer treatment service in the hospital. We believe this is necessary to guide cancer control programs of the country and contributes in awareness creation about the magnitude of the current and future cancer burden among policy makers, the general public, and international private or public health agencies.

## Methodology

### Study setting

Wolaita Sodo University Teaching referral Hospital is found in southern region of Ethiopia, 317 km from the capital Addis Ababa. It serves a total population of around 10 million. It has clinical services in different departments such as internal medicine, surgery, gynecology and obstetrics, pediatrics. There is also a pathology department with FNAC (fine needle aspiration cytology). Biopsy samples from the hospital are analyzed at a nearby private diagnostic centre and results are sent back to the hospital. Patients suspected of cancer used to be referred to the only oncology centre in the country at Addis Ababa. Since January 2021, a dedicated cancer wing with its own out patients and chemotherapy ward service has started giving services. Adult Patients from both surgical and medical departments evaluated and those suspected of cancer will be assigned to the newly opened cancer treatment wing where further evaluation and treatment is carried out.

### Study design & population

This was a retrospective record analysis of pathology service results. All patients with a record of diagnosis as cancer according to the pathology (FNAC and biopsy) result from Jan 2021 to Jun 2021 were included.

### Data collection and handling

Data was collected from the pathology department record books by physician working in the department. It was filtered by the investigator and entered in IBM SPSS version 22 (Chicago IL USA) and descriptive analysis was done. All patients included were those whose cancer diagnosis was made after establishment of new cancer treatment department in Wolaita Sodo University Teaching referral Hospital.

### Ethical approval

The conduction of this retrospective epidemiological study was approved approved by the Wolaita Sodo University referral Hospital Institutional Review Board. Since we are reporting a retrospective study of medical records and all data are fully anonymized, the ethics committee have waived the requirement for informed consent both for adults and minors datasets.

## Results

From January, 2021 to June, 2021 a total of 1,810 specimens (617 biopsies and 1,193 FNAC) were evaluated in department of pathology, Wolaita Sodo University Teaching referral Hospital. Of these 354 (19.5%) were found to be cancers. The median age of patients at diagnosis was 40 years, with range of 2–90 years. Of 354 malignant tumors, 221 (62.4%) were females and the 133 (37.6%) were males, with M:F ratio of 1:1.6. When we see the age distribution 336 (95%) occurred in adults age greater than 14 years and 18 (5%) occurred in children (Table 1).

**Table 1:** Sociodemography.

Sociodemography		Frequency N (%)
Sex	Male	133 (37.6%)
	Female	221 (62.4%)
Age (years)	0–14	18 (5%)
	15–24	30 (8.4%)
	25–54	225 (63.5%)
	55–64	50 (14.1%)
	≥65	31 (8.7%)

Age and sex distribution of cancer patients seen at pathology Department, Wolaita Sodo University hospital 2021.

Breast cancer accounts for 25.4% of the total cancer cases, out of this 1.6% (6 cases) are male breast cancers. Most of the patients are young, with median age of 35 years, ranging from 23–85 years (Table 2). Sarcomas accounts for 16.4% of all cancer cases, which made it the second most common of all cancers. Out of these 84.5% of them are soft tissue sarcomas and the rest 15.5% are bone sarcomas. Cancer of uteri cervix is the third commonest cancer according to this study, it comprises for 7.3% of all cases. But cervical cancer is the second top cancer in female adults above age of 14 years old.

**Table 2:** Commonest cancer types.

Cancer Type	Frequency
Breast Cancer	90 (25.4)
Soft tissue Sarcoma	49 (13.8)
Cancer of uteri cervix	26 (7.3)
Non Melanomatous Skin cancer	24 (6.8)
Non-Hodgkin lymphoma	23 (6.5)
Thyroid Cancer	17 (4.8)
Colorectal Cancer	15 (4.2)
HNSCC	12 (3.4)
Germ Cell tumor	11 (3.1)
Bone Sarcoma	9 (2.5)

The top 10 commonest cancer types seen at pathology department, Wolaita Sodo University Hospital of all age groups, 2021.

### The distribution of cancer by sex

**Fig 1:**
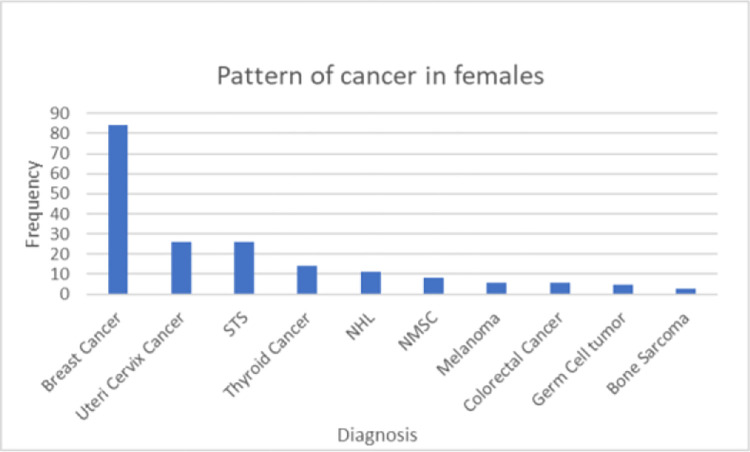
Cancer distribution pattern in females at Wolaita Sodo University Hospital, 2021. NMSC—Non Melanomatous Skin cancer, NHL- Non-Hodgkin lymphoma, STS—Soft tissue sarcoma.

**Fig 2:**
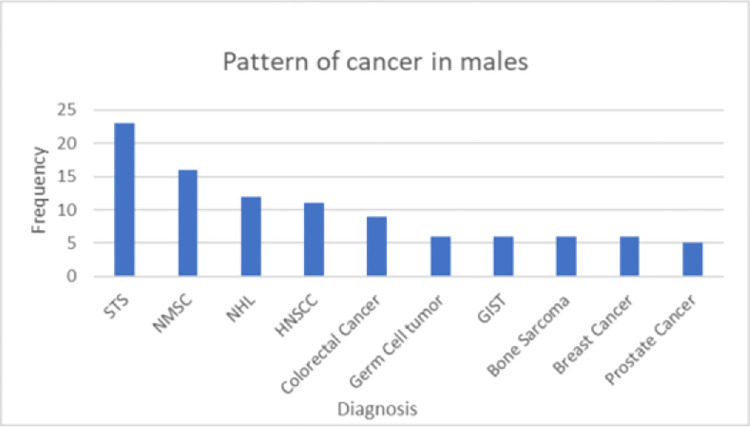
Cancer distribution pattern in males at Wolaita Sodo University Hospital, 2021. NMSC—Non Melanomatous Skin cancer, NHL- Non-Hodgkin lymphoma, STS—Soft tissue sarcoma. HNSCC—Head and Neck squamous cell cancer, GIST—Gastrointestinal stromal tumor.

The five commonest cancers in females are breast cancer, Cancer of uteri cervix, soft tissue sarcomas, thyroid cancer and non hodgkin lymphomas. The frequency distribution of female cancer is shown (Fig 1). In males, soft tissue sarcomas, non melanomatous skin cancers, non hodgkin lymphomas, head and neck squamous cell carcinomas (HNSCC) and colorectal cancer are the five commonest cancers (Fig 2).

### Cancer distribution pattern by age

**Fig 3:**
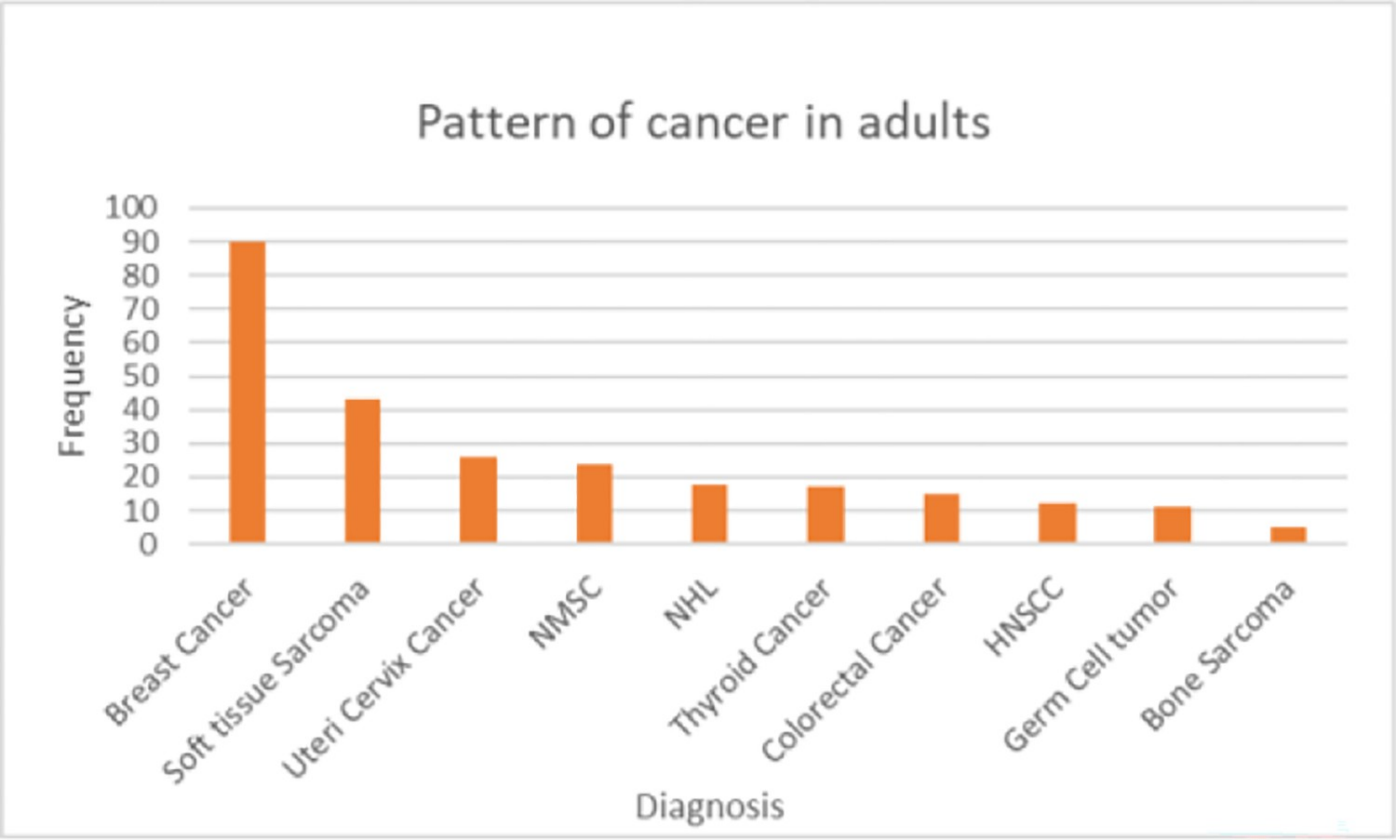
Cancer distribution pattern in adults in Wolaita Sodo University Hospital, 2021. NMSC—Non Melanomatous Skin cancer, NHL- Non-Hodgkin lymphoma. HNSCC—Head and Neck squamous cell cancer.

In adults of age greater than 14 years; Breast cancer, which accounts for 90 cases (26.8%), is the leading malignancy. Soft tissue sarcomas 43 (12.8%), Cancer of uteri cervix 26 (7.3%), were the other top malignancies following breast cancer (Fig 3). The three commonest childhood malignancies found in this study were Lymphomas, Soft tissue sarcomas and bone sarcomas (Table 3). In this study, age cut point of 14 years inclusive was considered as child.

**Table 3:** Pediatric common cancer types.

Cancer Type	Frequency
Lymphomas	7 (38.8%)
Soft tissue sarcoma	6 (33.3%)
Bone sarcoma	4 (22.2%)
Other	1 (5.5%)

The commonest cancer types seen pediatric age patients at pathology department, Wolaita Sodo University Hospital of all, 2021.

## Discussion

The GLOBOCAN 2020 report shows the annual incidence of new cases of cancer in Ethiopia has increased compared to previous reports [1]. In line with this report, the findings of this first study in a hospital in the southern region of Ethiopia indicates cancer is one of the major emerging health problems in Wolaita Sodo University Hospital. Different studies attribute such larger increase of cancer incidence in transitioning countries to a demographic changes further exacerbated by increasing risk factors associated with globalization and a growing economy [3]. Among the samples underwent histopathological examinations at department of pathology during the study period, one fifth of them were found to be a cancer. The majority of this histopathologically confirmed cancer diagnoses were made in females(62.8%) with median age of 40 years. Similar finding was reported from Addis Ababa cancer registry and other African countries where the majority of registered cancer cases were in women with highest incidence rate in the age group of 30 to 49 years [8, 9]. Such sex and age preferences may be explained by the occurrences of different female reproductive organ related malignancies due to repeated exposure to a different risk factors during earlier reproductive age [9].

The most common cancers in females were breast cancer (38%) followed by cancer of uteri cervix (11.7%). These top two cancer types reported by this study concurred with the Addis Ababa cancer registry report where the most common cancers in females were cancer of the breast (31.5%), followed by cancer of uteri cervix (14.1%) [6]. Findings from other cancer registries in Sub-Saharan Africa also showed Female breast and cancer of uteri cervix were the leading cancers among females [10]. However, hospital based study from University of Gondor hospital in Northern Ethiopia reported cancer of the cervix uteri is the commonest malignancy quoting the absence of well-functioning cervical cancer screening program as well as different life style of the population from the capital city Addis Ababa [7]. Although as with the other reports variables that directly measure cancer associated risk factors were not collected in our study too, westernized life style, urbanization, and stressed social factors might have contributed for the increased breast cancer ratio in this region [11, 12].

Interestingly, In males the leading types noted in this study were soft tissue sarcomas (17.3%) followed by non melanomatous skin cancers (12%) and non hodgkin lymphomas (9%). Soft tissue sarcomas are a rare and heterogeneous group of malignant tumors of mesenchymal origin that comprise less than 1 percent of all adult malignancies [13, 14]. Although most soft tissue sarcoma cases have no clearly defined etiology, some of the identified predisposing factors include genetic predisposition, exposure to radiation, chemical carcinogens, chronic irritation, Viruses (HHV,EBV & HIV) and lymphedema [15]. But, as mentioned above variables that directly measure cancer associated risk factors were not collected in our study and the predominance of soft tissue sarcoma cases was an unexpected finding and appears different finding from other similar studies in Ethiopia as well as other sub-saharan Africa reports. Most sub Saharan countries reported prostate cancers as leading cancer in males, whereas the Addis Ababa cancer registry reported colorectal cancer as the leading type of cancer, followed by prostate cancer [6]. Even though bone and soft tissue sarcoma were not segregated, a hospital based study from Tikur Anbassa hospital in Addis Ababa has reported sarcomas as the leading cancer type in males [16]. Thus the concordance of our study’s result with this other hospital based report invokes a new insight on the burden of sarcoma on Ethiopian male cancer patients. However, since our report is based solely on pathology specimens result, one plausible explanation for this high burden of soft tissue sarcoma might be the accessibility of tissue for FNAC and biopsy which could result in selection of patients with accessible site to be referred to pathology department. Moreover, the unavailability of services like, GI endoscopies, pulmonology or image guided biopsy procedures in the hospital might have resulted in lower incidence of tumors located in the other deep seated organs. However, this should still be confirmed with a further studies.

The study has several strengths and limitations. First, cancer diagnoses were histopathologically confirmed as opposed to diagnoses made solely clinically. Second, the study was conducted at a referral hospital in the southern region of the country as opposed to previous studies which are mainly concentrated in the capital city. One of the study limitations is the fact that it was done with a limited sample size over a shorter time-period. On the other hand, the included patients are those who were able to have a biopsy/FNA done, which depends on the availability of equipment and technical expertise in a hospital with limited resource. These limitations could underestimate certain types of cancers and should be considered when interpreting the study findings. Finally the unavailability of dedicated pediatric oncology wing might have resulted in a small proportion (5%) of pediatric cancer cases making it difficult to make further interpretations.

## Conclusion

This first study on cancer pattern at a hospital in southern part of Ethiopia indicates cancer is one of the common finding from histopathology samples analyzed at the hospital. In general, the patterns seen were similar to those reported in other regions of the country as well as in neighboring countries, where majority of the cases are in females of reproductive age group. Particular attention needs to be paid to this issue since this could have an impact on the economy of the country. Moreover, we found cancers with effective screening tests, such as cancer of uteri cervix and breast cancers are common in the hospital. Thus, timely access to preventive care along with effective educational and screening strategies is needed in Ethiopia to detect and treat cancer early. Comprehensive demographic and clinical data using population or facility based cancer registry is required to get better information for planning and monitoring cancer pattern in the region. Finally, our finding of higher proportion of soft tissue sarcomas both in males and females of all age groups in this region is disparate and requires further investigation.
